# Using a Large Language Model for Breast Imaging Reporting and Data System Classification and Malignancy Prediction to Enhance Breast Ultrasound Diagnosis: Retrospective Study

**DOI:** 10.2196/70924

**Published:** 2025-06-11

**Authors:** Su Miaojiao, Liang Xia, Zeng Xian Tao, Hong Zhi Liang, Cheng Sheng, Wu Songsong

**Affiliations:** 1 Department of Ultrasound, Fujian Provincial Hospital, Affiliated Hospital of Fuzhou University, 134 Dong Street, Gulou District, Fuzhou, 350500, China, 86 15960191609

**Keywords:** large language models, BI-RADS, breast ultrasound, ChatGPT-4, image-to-text–LLM, Breast Imaging Reporting and Data System

## Abstract

**Background:**

Breast ultrasound is essential for evaluating breast nodules, with Breast Imaging Reporting and Data System (BI-RADS) providing standardized classification. However, interobserver variability among radiologists can affect diagnostic accuracy. Large language models (LLMs) like ChatGPT-4 have shown potential in medical imaging interpretation. This study explores its feasibility in improving BI-RADS classification consistency and malignancy prediction compared to radiologists.

**Objective:**

This study aims to evaluate the feasibility of using LLMs, particularly ChatGPT-4, to assess the consistency and diagnostic accuracy of standardized breast ultrasound imaging reports, using pathology as the reference standard.

**Methods:**

This retrospective study analyzed breast nodule ultrasound data from 671 female patients (mean 45.82, SD 9.20 years; range 26‐75 years) who underwent biopsy or surgical excision at our hospital between June 2019 and June 2024. ChatGPT-4 was used to interpret BI-RADS classifications and predict benign versus malignant nodules. The study compared the model’s performance to that of two senior radiologists (≥15 years of experience) and two junior radiologists (<5 years of experience) using key diagnostic metrics, including accuracy, sensitivity, specificity, area under the receiver operating characteristic curve, *P* values, and odds ratios with 95% CIs. Two diagnostic models were evaluated: (1) image interpretation model, where ChatGPT-4 classified nodules based on BI-RADS features, and (2) image-to-text–LLM model, where radiologists provided textual descriptions, and ChatGPT-4 determined malignancy probability based on keywords. Radiologists were blinded to pathological outcomes, and BI-RADS classifications were finalized through consensus.

**Results:**

ChatGPT-4 achieved an overall BI-RADS classification accuracy of 96.87%, outperforming junior radiologists (617/671, 91.95% and 604/671, 90.01%, *P*<.01). For malignancy prediction, ChatGPT-4 achieved an area under the receiver operating characteristic curve of 0.82 (95% CI 0.79‐0.85), an accuracy of 80.63% (541/671 cases), a sensitivity of 90.56% (259/286 cases), and a specificity of 73.51% (283/385 cases). The image interpretation model demonstrated performance comparable to senior radiologists, while the image-to-text–LLM model further improved diagnostic accuracy for all radiologists, increasing their sensitivity and specificity significantly (*P*<.001). Statistical analyses, including the McNemar test and DeLong test, confirmed that ChatGPT-4 outperformed junior radiologists (*P*<.01) and showed noninferiority compared to senior radiologists (*P*>.05). Pathological diagnoses served as the reference standard, ensuring robust evaluation reliability.

**Conclusions:**

Integrating ChatGPT-4 into an image-to-text–LLM workflow improves BI-RADS classification accuracy and supports radiologists in breast ultrasound diagnostics. These results demonstrate its potential as a decision-support tool to enhance diagnostic consistency and reduce variability.

## Introduction

In recent years, large language models (LLMs) have advanced rapidly, demonstrating significant potential in health care. Their diverse applications have garnered considerable attention [1-4], driving revolutionary progress across various fields, including medicine [5]. LLMs are built upon large-scale datasets and continuously refined through iterative training, enabling them to handle a wide range of tasks, including medical diagnosis. LLMs provide diagnostic recommendations by processing key inputs, extracting relevant information, and performing associative reasoning [6]. In medical imaging analysis, the integration of LLMs with image-text conversion models enables the creation of a continuous interactive system that seamlessly integrates with clinical practice, delivering more precise and personalized health care services [7]. This approach enhances medical technology, broadens clinical applications, and partially alleviates the challenges posed by limited health care resources. GPT-4, developed by OpenAI, is one of the most widely recognized LLMs. Although its applications in medical imaging analysis have shown promise, the specific role and diagnostic accuracy of LLMs in this domain remain insufficiently explored. Initial research on LLMs primarily focused on evaluating their language generation capabilities. Most discussions surrounding LLMs, particularly ChatGPT, have revolved around comments, replies, or question-and-answer formats [8-11]. The findings have been promising [12]. However, many studies have primarily focused on response evaluation rather than quantitative assessments of diagnostic accuracy and have lacked reference standards such as pathology or laboratory examinations [13]. In addition, the ability of LLMs to generate structured diagnostic reports, such as Breast Imaging Reporting and Data System (BI-RADS) classifications, has not been systematically evaluated, raising concerns about their reliability in clinical decision-making. Furthermore, LLMs may inadvertently perpetuate existing biases and disparities. Therefore, to address this gap, it is essential to demonstrate the application of LLMs in medical imaging. Breast cancer serves as an optimal case study for this purpose due to its high incidence, critical need for early detection, and reliance on imaging for diagnosis. Breast cancer is one of the most life-threatening diseases, second only to lung cancer [14,15]. The likelihood of breast cancer survival is largely determined by the stage at which it is detected, which significantly impacts chemotherapy outcomes [16-18]. Early diagnosis and effective treatment of breast cancer can significantly reduce mortality rates [19]. Unlike lung cancer, which primarily relies on computed tomography (CT）imaging, or neurological disorders, which depend on magnetic resonance imaging (MRI) and functional imaging, breast cancer diagnosis heavily incorporates ultrasound due to its accessibility, cost-effectiveness, and suitability for dense breast tissue—particularly in younger patients and in regions with high breast cancer incidence, such as China [20]. Therefore, in China, ultrasound has become the preferred imaging modality for breast lesion screening and preoperative evaluation [21]. Breast ultrasound not only facilitates routine detection of breast cancer but also aids in resolving inconclusive mammographic findings [22-24]. The BI-RADS, proposed by the American College of Radiology (ACR), is a standardized system designed to describe breast imaging findings [25]. It assists radiologists and breast surgeons in standardizing the assessment of malignancy probability in breast lesions and in guiding subsequent diagnostic and treatment plans. Physicians classify breast lesions based on specific characteristics, including shape, orientation, margins, echogenic pattern, posterior features, and the presence of calcifications and associated findings. However, unlike structured reporting systems in CT-based lung cancer screening or MRI-based neurological assessments, BI-RADS assessment in breast ultrasound remains highly dependent on operator experience. This reliance leads to interobserver variability and diagnostic inconsistency, as radiologists with different levels of expertise may provide varying interpretations. In addition, ultrasound evaluation requires extensive training and certification, making it resource-intensive and subject to human variability.

Given these challenges, breast ultrasound serves as an ideal testbed for evaluating LLM-assisted imaging interpretation. First, the structured nature of BI-RADS makes it well-suited for artificial intelligence (AI)-driven standardization, potentially reducing subjective variability. Second, the widespread clinical use of ultrasound in breast cancer detection allows for large-scale validation of LLM-generated reports against pathology results. Unlike lung CT or brain MRI, where AI-based segmentation and feature extraction already play a significant role, breast ultrasound interpretation lacks a robust AI-driven standardization framework, highlighting the potential value of LLM integration. LLMs offer a promising solution by providing standardized interpretations, reducing interobserver variability, and assisting in structured reporting. Their ability to generate BI-RADS reports based on ultrasound findings suggests a potential role in improving diagnostic consistency and streamlining clinical workflows. Despite these advantages, the reliability and diagnostic accuracy of LLM-generated BI-RADS assessments remain unclear.

To bridge this research gap, this study aims to systematically evaluate the diagnostic performance, consistency, and clinical utility of mainstream LLMs in breast nodule assessment. By comparing LLM-generated BI-RADS categorizations with pathology-confirmed diagnoses, we seek to determine whether LLMs can enhance reproducibility, improve standardization, and assist radiologists in breast cancer diagnosis.

## Methods

### Study Design

To compare the diagnostic performance of the LLMs and radiologists with different levels of experience in interpreting breast ultrasound images. First, among the widely used LLMs, ChatGPT, and Gemini are prominent. However, since Gemini does not support image analysis, we selected OpenAI’s ChatGPT-4 for this study [26], using the BI-RADS standard for diagnosing breast nodules. For clarity, while LLMs refer to a broad category of AI models, this study specifically focuses on ChatGPT-4. We analyzed the accuracy of ChatGPT-4 in classifying breast nodules using the BI-RADS system and assessed its diagnostic performance in distinguishing between benign and malignant breast nodules. In subsequent analyses, the performance of two different models was evaluated: image-to-text–LLM model and image interpretation model.

#### Image-to-Text–LLM Model

**Figure 1.**
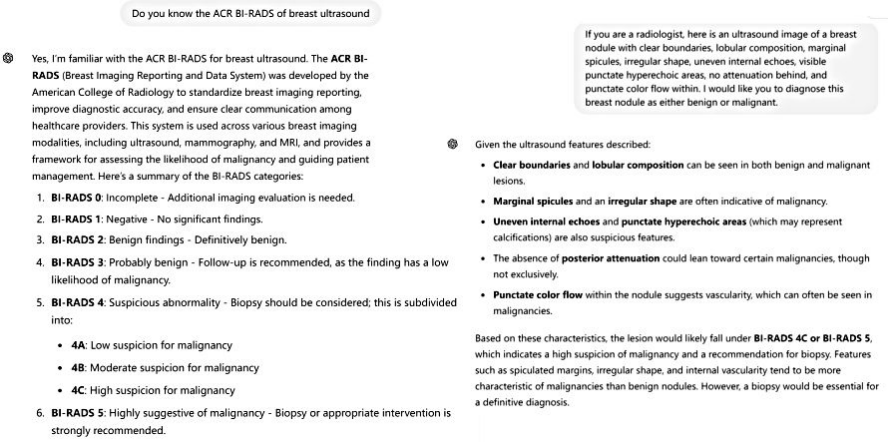
The screenshot demonstrates ChatGPT-4 (OpenAI; ) analyzing a single breast nodule in image-to-text–large language model. This response was recorded as a malignant diagnosis.

Radiologists first interpreted the ultrasound images and generated textual descriptions using a standardized BI-RADS reporting template. This template included predefined descriptors for nodule shape, margin, echogenicity, and other key ultrasound features. ChatGPT-4 then analyzed the text and determined whether the nodule was benign or malignant based on the extracted keywords (Figure 1). To minimize variability in descriptions, all radiologists were trained in the standardized reporting format, and their reports were randomly reviewed by a senior radiologist to ensure consistency.

#### Image Interpretation Model

**Figure 2.**
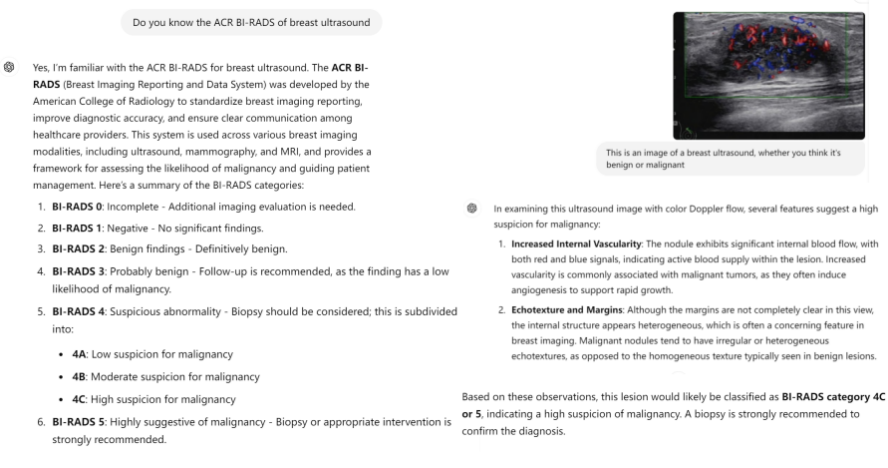
The screenshot demonstrates ChatGPT-4 (OpenAI; analyzing a single breast nodule in image interpretation model. This response was recorded as a malignant diagnosis.

The ultrasound images were provided directly, and the model performed benign or malignant diagnosis based on BI-RADS classification and image analysis (Figure 2). All radiologists were blinded to the pathological results. The radiologists were divided into two senior and two junior groups. The senior radiologists each had over 15 years of experience in breast ultrasound, while the junior radiologists had less than 5 years of experience.

The BI-RADS classifications were determined by consensus between two senior radiologists. If their conclusions differed, a third senior radiologists (WSS, with 21 years of experience in breast ultrasound) was consulted to reach a final decision through discussion.

### Patient Data Collection

**Figure 3.**
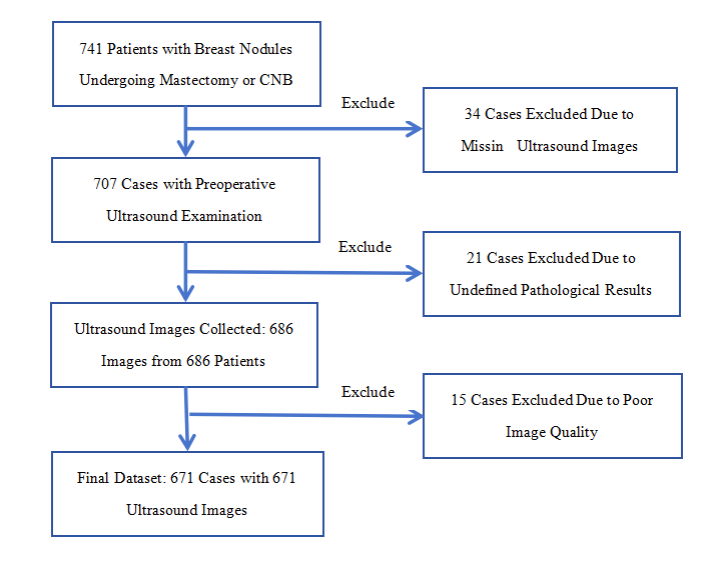
Flowchart of patient inclusion, exclusion, and study enrollment. CNB: core needle biopsy

Retrospective collection of ultrasound images of breast nodules diagnosed through biopsy or surgical excision at Fujian Provincial Hospital, Fuzhou University Affiliated Hospital, between June 2019 and June 2024 (385 benign and 286 malignant cases). Patients without ultrasound examinations, those with low-quality images, or those with inconclusive pathological results were excluded (Figure 3). Each image corresponded to an individual breast nodule. For nodules suspected to be benign on biopsy, follow-up of at least 6 months was required. All lesions were ultimately classified as benign or malignant based on histopathological results.

### Evaluation Index

**Figure 4.**
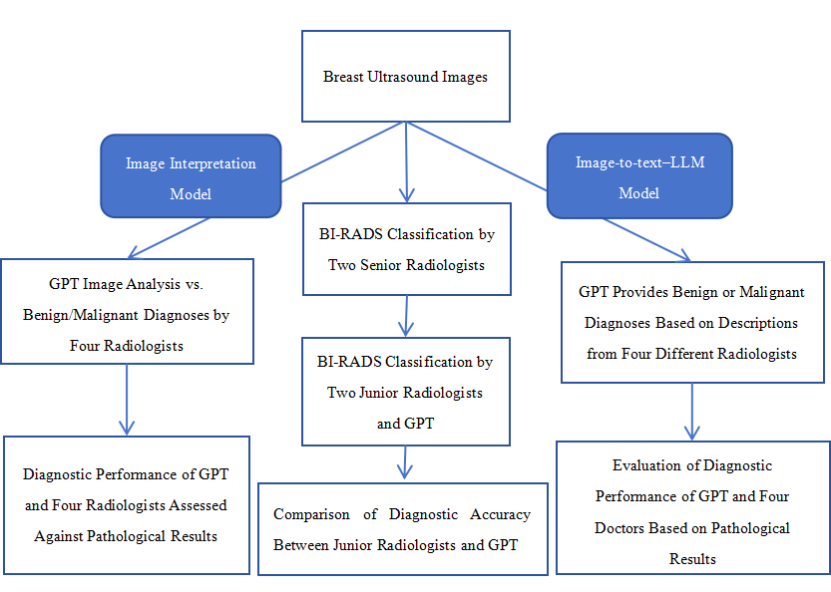
Study Design. LLM: large language model; BI-RADS: Breast Imaging Reporting and Data System.

The diagnostic performance of ChatGPT-4 in two different models (Image-to-text–LLM model and image interpretation model) was compared with that of radiologists with varying levels of experience. The analysis included the accuracy of BI-RADS classifications by junior radiologists and the calculation of key performance metrics, including sensitivity, specificity, accuracy, and the area under the receiver operating characteristic curve (AUC) with 95% CIs (Figure 4).

### Statistical Analysis

All statistical analyses were performed using SPSS software (IBM, version 20.0) and MedCalc (MedCalc Software, version 12.7). Continuous data were expressed as mean (SD), and categorical data were presented as frequencies and percentages. The analysis included the calculation of key performance metrics, including accuracy, sensitivity, specificity, and the AUC with 95% CIs. Finally, McNemar’s test and DeLong’s test were used to compare the diagnostic performance of ChatGPT-4 under different deployment strategies. A *P* value of <.05 was considered statistically significant.

### Ethical Considerations

This study was approved by the Institutional Review Board of Fujian Provincial Hospital. Informed consent was waived due to the retrospective design. No identifiable patient information was shared with the LLMs. This study is a retrospective study, and informed consent was waived due to its retrospective design. No patient privacy information was provided to the LLMs.

## Results

### Patient Characteristics

A total of 671 female patients were included in the study (mean 45.82, SD 9.20 years; range 26‐75 years). A total of 671 ultrasound images of breast nodules were collected, including 385 benign cases (57.38%) and 286 malignant cases (42.62%), with 428 nodules on the left side and 243 on the right side (Table 1). Pathological diagnoses within 3 months after ultrasound examination were based on surgical resection or core needle biopsy. Patients with benign pathology from core needle biopsy were followed up for an average of approximately 11.2 months. The inclusion and exclusion criteria for patients and images are detailed in Figure 3.

**Table 1.** Demographic and clinical characteristics of patients. Unless otherwise specified, the data represents the number of survey results, with percentages in parentheses.

Characteristic	Values
Age (years)^a^, mean (SD), range	45.82 (9.20; 26‐75）
Location	
Left	428 (63.79）
Right	243 (36.21）
Nodule pathology	
Benign	385 (57.38）
Malignant	286 (42.62）

a Data are presented as mean (SD), with the range in parentheses.

### Representation of ChatGPT-4 in Classification

As shown in Table 2, the BI-RADS classifications for the 671 ultrasound images include categories BI-RADS 3, 4 (A-C), and 5. The overall accuracy of the ChatGPT-4 in identifying BI-RADS classifications was 96.87% (650/671), surpassing junior radiologist 1 (617/671, 91.95%) and junior radiologist 2 (604/671, 90.01%). Notably, the model demonstrated significant differences in classification performance compared to both junior radiologists for BI-RADS 4A and to junior radiologist 2 for BI-RADS 3 and 4C.

**Table 2.** The effectiveness of ChatGPT-4 in classification. The *P* value represents the comparison with ChatGPT-4

Classification	N	ChatGPT-4, n (%)	Junior physicians 1, n (%)	*P* value	Junior Physicians 2, n (%)	*P* value
BI-RADS^a^ 3	93	90（96.77）	86 (92.47)	.29	82 (88.17)	.04
BI-RADS 4A	374	361 (96.52)	345 (92.25)	.02	341 (91.18)	<.01
BI-RADS 4B	98	95 (96.94)	91 (92.86)	.22	90 (91.84)	.13
BI-RADS 4C	56	55 (98.21)	50 (89.29)	.06	47 (83.93)	<.01
BI-RADS 5	50	49 (98.00)	45 (90.00)	.13	44 (88.00)	.06

a BI-RADS: Breast Imaging Reporting and Data System.

### Performance of the Image Interpretation Model in Predicting Benign and Malignant Breast Nodules

As shown in Table 3, senior radiologist 1 demonstrated excellent performance in diagnosing benign and malignant breast lesions via ultrasound, with an AUC of 0.85 (95% CI 0.81‐0.88), an accuracy of 83.16% (558/671 cases), a sensitivity of 91.61% (262/286 cases), and a specificity of 77.40% (298/385 cases). ChatGPT-4 exhibited comparable performance to the image interpretation model of both senior radiologists in terms of accuracy, sensitivity, and specificity. Notably, it outperformed senior radiologist 2 in AUC, with the difference being statistically significant (*P*<.01). The image interpretation model showed diagnostic performance comparable to that of the 2 senior radiologists, particularly achieving a sensitivity of 90.56% (259/286 cases). Furthermore, it consistently outperformed the predictive models of the junior radiologists in AUC, accuracy, and sensitivity (*P*<.01), while showing a statistically significant difference in specificity compared to junior radiologist 1 (*P*<.01).

**Table 3.** The performance of image interpretation model in predicting benign and malignant breast nodules. Except were indicated, numbers in parentheses are numbers of nodules.

Strategy	AUC^a^,^b^ (95% CI)	*P* value	Accuracy, n (%), Total=671	*P* value	Sensitivity, n (%), Total=286	*P* value	Specificity, n (%), Total=385	*P* value
ChatGPT-4	0.82 (0.79-085）	—^c^	541 (80.63)	—	259/286 (90.56)	—	73.51(283/385)	—
Senior Radiologist 1	0.81 (0.77-0.84）	.10	536 (79.88)	.88	246/286 (86.01)	.18	75.32(290/385)	.50
Senior Radiologist 2	0.79 (0.76-0.83）	<.01	524 (78.09)	.24	248/286 (86.71)	.23	71.69(276/385)	.63
Junior Radiologist 1	0.66 (0.62-0.70）	<.01	440 (65.57)	<.01	181/286 (63.33)	<.01	67.27(259/385)	<.01
Junior Radiologist 2	0.67 (0.62-0.71）	<.01	446 (66.47)	<.01	191/286 (66.78)	<.01	66.23(255/385)	.13

a Data in parentheses for the AUC metric are 95% CIs.

b AUC: area under a receiver operating characteristic curve.

c Not applicable

### Performance of ChatGPT-4 in Predicting Benign and Malignant Breast Nodules Based on Image-To-Text–LLM Model

As shown in Table 4, the image interpretation model of ChatGPT-4 yielded an AUC of 0.82 (95% CI 0.79‐0.85), an accuracy of 80.63% (541/671 cases), a sensitivity of 90.56% (259/286 cases), and a specificity of 73.51% (283/385 cases). Under the image-to-text–LLM model, ChatGPT-4 demonstrated performance comparable to the 2 senior radiologists while surpassing the models of junior radiologists in AUC, accuracy, sensitivity, and specificity (*P*<.001). The four radiologists with varying levels of experience showed improvements in AUC, accuracy, sensitivity, and specificity after image-to-text–LLM model compared to image-to-text–LLM model.

**Table 4.** The performance of ChatGPT-4 in predicting benign and malignant breast nodules based on image-to-text–large language model. Note.—Except were indicated, numbers in parentheses are numbers of nodules.

Diagnostic performance	ChatGPT-4	Senior radiologist 1	Senior radiologist 2	Junior radiologist 1	Junior radiologist 2
AUC^a^, ^b^ (95% CI)	0.82 (0.79-085）	0.85 (0.81-0.88）	0.82 (0.78-0.85）	0.64 (0.62-0.70）	0.63 (0.59-0.67）
*P* value	—^c^	.10	.77	<.01	<.01
Accuracy, n (%) Total=671	541 (80.63)	558 (83.16）	546 (81.37）	486 (72.43）	463 (69.00）
*P* value	—	.13	.72	<.01	<.01
Sensitivity, n (%) Total=286	259 (90.56)	262 (91.61)	248 (86.71)	240 (83.92)	212 (74.13)
*P* value	—	.87	.29	.02	<.01
Specificity, n (%) Total=385	283 (73.51)	298 (77.40)	294 (76.36)	248 (64.42)	253 (65.71)
*P* value	—	.10	.18	<.01	.01

a Data in parentheses for the AUC metric are 95% CIs.

b AUC: area under a receiver operating characteristic curve

c Not applicable

## Discussion

### Principal Findings

In medical diagnostics, logical reasoning often forms the foundation of treatment, requiring higher-order cognitive skills such as application, analysis, and evaluation. Bhayana et al [8] demonstrated that ChatGPT nearly passed a radiology board-style exam despite lacking radiology-specific pretraining or direct access to imaging data. Despite the promising potential of LLMs in medical imaging [27], there is a lack of research on their feasibility in addressing reasoning tasks associated with medical diagnoses based on breast-specific reference standards, such as pathology. In this study, we used BI-RADS and standardized pathological reporting as reference standards, integrating LLMs into the diagnostic process to emphasize the benefits of combining ChatGPT-4 with image-to-text technologies. We validated the potential application of ChatGPT-4 in breast nodule ultrasound diagnostics, using pathological results as the benchmark to evaluate their performance in standardized reporting and diagnostic consistency. The results showed that ChatGPT-4 performed exceptionally well in BI-RADS classification and benign-versus-malignant predictions of breast nodules. Its diagnostic performance was significantly superior to that of less experienced radiologists. This lays a foundation for the practical application of LLMs in enhancing the standardization and accuracy of medical imaging diagnostics while providing evidence to support further exploration of their clinical utility. ChatGPT-4 achieved an overall accuracy of 96.87% in BI-RADS classification and an AUC of 0.82 for benign-versus-malignant predictions (AUC 0.82, 95% CI 0.79‐0.85). Its accuracy, sensitivity, and specificity were 80.63% (541/671), 90.56% (259/286), and 73.51% (283/385), respectively. Notably, ChatGPT-4 outperformed less experienced radiologists in BI-RADS categories 4A, 3, and 4C. This indicates that ChatGPT-4 can deliver highly consistent and accurate diagnostic results, reducing variability caused by limited experience. Integrating LLMs into breast condition classification can minimize diagnostic errors and ultimately improve patient outcomes significantly. LLMs can be trained to identify and interpret individual variations, ultimately assisting in the development of new classification standards. This approach may address the lack of universally accepted breast disease classifications. Furthermore, integrating image-to-text conversion by radiologists with ChatGPT-4 improved the AUC, accuracy, sensitivity, and specificity for all 4 physicians, with notable performance enhancements observed among less experienced radiologists. This underscores that while AI has made significant advancements in medical imaging and diagnostics, human expertise remains indispensable. While ChatGPT-4 demonstrated strong diagnostic capabilities, it is important to compare its performance with existing AI-driven ultrasound diagnostic models. Prior deep-learning models have shown comparable or even superior performance in specific ultrasound tasks. For example, convolutional neural networks (CNNs) and transformer-based models trained on large annotated datasets have achieved AUCs exceeding 0.90 in distinguishing benign from malignant breast lesions [28,29]. These models directly analyze imaging data, extracting pixel-level features for classification. In contrast, ChatGPT-4 processes structured text inputs rather than images, highlighting a key trade-off between interpretability and diagnostic precision. While CNNs and other deep-learning models operate as “black-box” systems with high accuracy, LLMs like ChatGPT-4 provide enhanced transparency and reasoning, allowing clinicians to trace the logic behind their decisions. Thus, future studies should explore hybrid approaches that integrate deep-learning image analysis with LLM-driven reasoning to maximize diagnostic performance. Our study, grounded on pathological findings as the reference standard, provides substantial evidence that using pathology-based benchmarks to evaluate LLMs in ultrasound diagnostic reasoning and information processing differs significantly from previous approaches. Earlier studies primarily relied on expert questionnaires and subjective judgments to assess LLMs’ text comprehension, summarization, and generation abilities in the context of medical imaging reports or inquiry responses [30,31]. The results showed that ChatGPT-4, when integrated with clinician-led image-to-text conversion strategies, achieved optimal performance. This demonstrates that LLMs can effectively assist radiologist, particularly less experienced radiologists, thereby improving overall diagnostic quality. Compared to senior radiologists, ChatGPT-4 demonstrated sensitivity (90.56%) in benign-versus-malignant diagnosis of breast nodules that closely matched that of 2 senior radiologists (91.61% and 86.71%). However, its specificity was slightly lower (73.51% vs 77.40% and 76.36%). These findings suggest that ChatGPT-4’s diagnostic reasoning, when provided with structured textual inputs, approaches that of senior radiologists. However, its performance remains constrained by its reliance on human-generated descriptions, rather than direct image interpretation. Unlike black-box deep-learning models, ChatGPT-4 offers a transparent reasoning process, enhancing trust in AI-assisted diagnostics. This feature is particularly valuable in clinical practice and medical education, as it enables junior radiologists to follow structured diagnostic logic rather than relying on uninterpretable AI-generated outputs. By standardizing ultrasound diagnostic reasoning, ChatGPT-4 could improve the consistency and quality of radiological assessments, especially among less experienced practitioners. This study demonstrates the advantages of LLMs in breast ultrasound diagnosis. First, LLMs standardize the diagnostic process, significantly reducing inconsistencies caused by variations in physician experience, making them particularly beneficial for less experienced radiologists. Second, they offer high efficiency in processing large image datasets, alleviating the workload of radiologists, particularly in breast cancer screening programs. Furthermore, LLMs can be incorporated into standardized reporting frameworks such as BI-RADS, providing real-time decision support to optimize clinical workflows. Rather than replacing CNN-based models, ChatGPT-4 functions as a complementary tool that interprets structured radiological descriptions, offering an alternative diagnostic strategy. Most traditional diagnostic support software focuses on extracting imaging features rather than providing comprehensive diagnostic suggestions. Our study paves the way for applying LLMs in medical image-based diagnostic reasoning. Future research should explore multimodal AI models that integrate ultrasound, CT, and MRI data to further enhance diagnostic accuracy. In addition, refining image-to-text conversion methods and reducing reliance on manual input will be critical for advancing fully automated diagnostic systems. Validating these findings on multicenter datasets is essential to ensure generalizability.

### Limitations

Despite demonstrating the potential of LLMs in ultrasound diagnostics, this study has certain limitations. First, it did not use the latest LLM versions, and ChatGPT-4 lacks the capability to directly interpret medical images. Its diagnostic process depends on structured text inputs provided by physicians, limiting its feasibility for fully automated diagnostics. Therefore, its current role is best suited as a decision-support tool rather than an independent diagnostic system. Second, the study was based on BI-RADS standards and did not account for complex imaging features or multimodal data, potentially restricting the model’s applicability to challenging cases. Third, the dataset was derived from a single institution, introducing potential selection bias. In addition, the patient cohort was drawn from a specific geographic and demographic background, which may not fully represent broader populations. Future studies should include diverse, multiethnic patient groups to improve external validity. Finally, while pathological results were used as the gold standard, they are not always available in routine clinical practice. Investigating alternative validation methods, such as long-term follow-up outcomes, could further enhance the clinical applicability of LLM-assisted diagnostics.

### Conclusion

This study systematically evaluates the application of LLMs in breast ultrasound diagnosis. The results show that ChatGPT-4 achieves diagnostic performance comparable to senior radiologists while significantly improving the accuracy of less experienced radiologists. These findings highlight its potential to enhance diagnostic consistency and standardization. As AI and imaging technologies advance, LLMs are expected to further improve diagnostic accuracy and efficiency. Future research should focus on multicenter validation and multimodal imaging integration to further assess its clinical applicability.

## Abbreviations

ACR: American College of Radiology
AI: artificial intelligence
AUC: area under the receiver operating characteristic curve
BI-RADS: Breast Imaging Reporting and Data System
CNN: convolutional neural network
CT: computed tomography
LLM: large language model
MRI: magnetic resonance imaging
